# Membrane Damage Elicits an Immunomodulatory Program in *Staphylococcus aureus*

**DOI:** 10.1371/journal.ppat.1000802

**Published:** 2010-03-12

**Authors:** Ahmed S. Attia, Meredith A. Benson, Devin L. Stauff, Victor J. Torres, Eric P. Skaar

**Affiliations:** 1 Department of Microbiology and Immunology, Vanderbilt University Medical Center, Nashville, Tennessee, United States of America; 2 Department of Microbiology and Immunology, Faculty of Pharmacy, Cairo University, Cairo, Egypt; 3 Department of Microbiology, New York University School of Medicine, New York, New York, United States of America; Harvard Medical School, United States of America

## Abstract

The *Staphylococcus aureus* HrtAB system is a hemin-regulated ABC transporter composed of an ATPase (HrtA) and a permease (HrtB) that protect *S. aureus* against hemin toxicity. *S. aureus* strains lacking *hrtA* exhibit liver-specific hyper-virulence and upon hemin exposure over-express and secrete immunomodulatory factors that interfere with neutrophil recruitment to the site of infection. It has been proposed that heme accumulation in strains lacking *hrtAB* is the signal which triggers *S. aureus* to elaborate this anti-neutrophil response. However, we report here that *S. aureus* strains expressing catalytically inactive HrtA do not elaborate the same secreted protein profile. This result indicates that the physical absence of HrtA is responsible for the increased expression of immunomodulatory factors, whereas deficiencies in the ATPase activity of HrtA do not contribute to this process. Furthermore, HrtB expression in strains lacking *hrtA* decreases membrane integrity consistent with dysregulated permease function. Based on these findings, we propose a model whereby hemin-mediated over-expression of HrtB in the absence of HrtA damages the staphylococcal membrane through pore formation. In turn, *S. aureus* senses this membrane damage, triggering the increased expression of immunomodulatory factors. In support of this model, wildtype *S. aureus* treated with anti-staphylococcal channel-forming peptides produce a secreted protein profile that mimics the effect of treating *ΔhrtA* with hemin. These results suggest that *S. aureus* senses membrane damage and elaborates a gene expression program that protects the organism from the innate immune response of the host.

## Author Summary

*Staphylococcus aureus* infects almost every tissue within the human body utilizing a range of virulence factors to combat host defenses. The expression of these virulence factors is a tightly regulated process; however, the signals sensed by *S. aureus* during infection remain elusive. It has been hypothesized that heme toxicity is a signal sensed by *S. aureus* during infection. This hypothesis is based on the observation that *S. aureus* mutants which are incapable of relieving heme-toxicity due to inactivation of the ATPase HrtA elicit an immunomodulatory program that interferes with neutrophil recruitment to the site of infection. In keeping with this, *S. aureus hrtA* mutants exhibit liver-specific hypervirulence. Herein, we provide evidence for an alternative model to explain the hypervirulent phenotype of *S. aureus ΔhrtA*. We demonstrate that instead of accumulation of heme toxicity being the trigger for the observed immunomodulatory program, dysregulated pore formation caused by the HrtB permease triggers the anti-neutrophil response. In support of this model, over-expression of HrtB in wildtype *S. aureus* or exposing *S. aureus* to channel-forming antimicrobial peptides induces a similar immunomodulatory program. Our work provides evidence that *S. aureus* senses membrane damage and induces an immunomodulatory circuit that helps the pathogen evade immune-mediated clearance.

## Introduction

*Staphylococcus aureus* is a Gram-positive commensal bacterium that colonizes the skin and anterior nares of approximately 25 % of the population [1]. Upon breaching these initial sites of colonization, *S. aureus* is capable of causing a range of infections [2]. Staphylococcal infections affect almost every organ in the human body ranging from minor skin and soft tissue infections to more serious diseases such as endocarditis, septicemia, pneumonia and toxic shock syndrome [3],[4]. In order to cause such a diverse array of pathologies, *S. aureus* employs an arsenal of virulence factors including proteins that contribute to immune evasion and alter immune system function [5].

During infection, *S. aureus* faces several barriers that interfere with its ability to replicate and colonize its host. One of these barriers is the paucity of free iron, which is a critical component of several reactions within the bacterial cell [6]. To circumvent this barrier, *S. aureus* can satisfy its iron needs through acquisition of the metalloporphyrin heme, which is a cofactor of host hemoglobin and myoglobin. *S. aureus* binds, transports, and releases heme into the cytoplasm through the combined action of the iron regulated surface determinant (Isd) system and the heme transport system (Hts) [7],[8],[9],[10],[11]. Although heme is a valuable nutrient iron source at low concentrations, high concentrations of heme are toxic and therefore heme acquisition necessitates the presence of heme detoxification systems. In this regard, *S. aureus* senses heme exposure through the HssRS two-component system [12],[13] resulting in the up-regulation of the heme regulated transporter (HrtAB). HrtAB is an ABC-type transporter that consists of an ATPase (HrtA) and a permease (HrtB) which work together to alleviate heme toxicity and protect the cell from the adverse effects of heme accumulation [13],[14].

ABC transporters represent one of the largest protein super-families in both eukaryotes and bacteria [15]. They play a pivotal role in the transport of a diverse group of molecules across the lipid bilayer of the cell membrane either to import nutrients or to export waste and toxic products [16]. ABC transporters consist of four domains; two transmembrane domains (TMD) and two nucleotide binding domains (NBD) that couple ATP hydrolysis to the transport of solutes across the membrane [17],[18]. The subcellular location of ABC transporters within the cell membrane makes it possible that alterations of ABC transporter structure or function induce membrane stress.

*S. aureus* strains lacking HrtA (*ΔhrtA*) exhibit liver-specific hyper-virulence in an animal model of infection suggesting that heme toxicity is linked to staphylococcal virulence. Consistent with this supposition, hemin-exposed *ΔhrtA* exhibits increased expression and secretion of several immunomodulatory proteins that are modeled to interfere with immune cell migration to the site of infection. Taken together, these findings have led to a model whereby *S. aureus* strains unable to alleviate heme toxicity through HrtAB activate an immunomodulatory program resulting in decreased killing by immune cells and increased virulence [13].

In this manuscript, we propose an alternative model to explain the hypervirulence of *ΔhrtA.* We report that the stimulus which leads to the increased expression of immunomodulatory proteins in hemin-exposed *ΔhrtA* is membrane damage caused by permease over-expression rather than intracellular hemin accumulation. Consistent with this, we present a series of data suggesting that permease expression in the absence of a cognate ATPase produces dysregulated pores in the membrane. This membrane damage is somehow sensed by *S. aureus* leading to increased expression and secretion of immunomodulatory factors. This model is further supported by the observation that exposing wildtype *S. aureus* to the channel forming antimicrobial peptide (AMP) gramicidin results in a secreted protein profile that mimics that of *ΔhrtA* exposed to hemin. The pathological relevance of this response is demonstrated by results reported here which show that up-regulation of secreted proteins is responsible for the hyperviurlence of strains lacking *hrtA*. Taken together, these data suggest a model whereby *S. aureus* senses pore-formation in the cell membrane to elicit an immunomodulatory program that interferes with neutrophil recruitment to the site of infection.

## Results

### *S. aureus ΔhrtA* increases expression of immunomodulatory proteins

**Figure 1:**
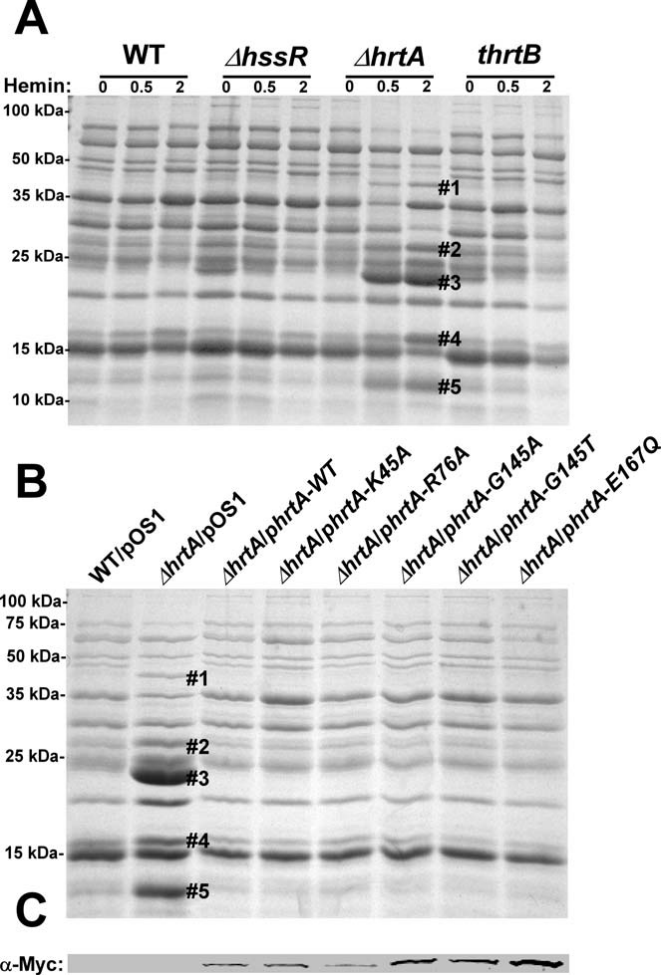
Up-regulation of immunomodulatory proteins is caused by loss of expression of HrtA. (A) Bacterial strains were grown in (0, 0.5 and 2 µM) hemin in RPMI/CAS for 18 hours and proteins in culture supernatants were precipitated using 10% TCA, separated using 15% SDS-PAGE, and stained with Coomassie blue. (B) Exoprotein profiles of wildtype transformed with control plasmid pOS1 and *ΔhrtA* transformed with control plasmid or plasmids expressing WT HrtA, catalytically inactive HrtA variants (K45A, G145A, G145T, and E167Q) or partially catalytically active HrtA (R76A) [14]. All strains were grown in the presence of 1 µM hemin. Proteins up-regulated under the indicated condition that have been identified previously using mass spectrometry are marked with a #. The predicted identities of the proteins in these bands are as follows; #1 (Exotoxin 8, SACOL0472), #2 (Exotoxin, SACOL0478), #3 (Exotoxin 3 and 5, SACOL0468/0473), #4 (Efb, SACOL1168), and #5 (FLIPr, SACOL1166). Positions of protein molecular mass markers in kilodaltons (kDa) are indicated on the left side of panels A and B. (C) Immunoblot analysis of protoplast lysates of strains analyzed in (B). Proteins were resolved using 15% SDS-PAGE, transferred to nitrocellulose membrane, probed with 9E10 anti-C-Myc monoclonal primary and AlexaFluor-680-conjugated anti-mouse secondary antibodies. Membranes were then dried and scanned using an Odyssey Infrared Imaging System.

In an effort to elucidate the mechanism responsible for the hypervirulence of *S. aureus ΔhrtA,* we analyzed the secreted protein profiles of staphylococcal strains with mutations in the Hss and Hrt systems. Upon exposure of *ΔhrtA* to hemin in concentrations of either 0.5 or 2 µM, up-regulation of the production and secretion of at least five proteins was observed (Fig. 1A). These proteins have been previously identified through mass spectrometry to be immunomodulatory proteins that interfere with neutrophil recruitment to the site of infection [13],[19],[20],[21]. This finding suggests that strains unable to alleviate hemin toxicity through HrtAB increase the expression of immunomodulatory proteins. However, a hemin-dependent increase in protein expression was not observed in either wildtype *S. aureus* or a strain lacking functional HssRS (*ΔhssR*), the regulatory system which controls hemin-dependent *hrtAB* expression (Fig. 1A) [12]. In addition, the secreted protein profile of a hemin-exposed *S. aureus* strain containing a transposon insertion that inactivates the permease HrtB (*thrtB*) closely resembled that of wildtype and did not show changes similar to those observed in *ΔhrtA* exposed to hemin (Fig. 1A). These data indicate that hemin-exposed *S. aureus ΔhrtA* increases the secretion of immunomodulatory proteins, but this immunomodulatory response does not occur upon loss of HrtB.

### The presence of a catalytically inactive HrtA prevents the hemin-dependent immunomodulatory response

One potential explanation for the discrepancy in secreted protein profiles observed between *ΔhrtA* and *thrtB* is that the *ΔhrtA* strain has accumulated secondary mutations that are responsible for altering the secretome. This possibility was eliminated by the demonstration that the secreted protein profile of hemin-exposed *ΔhrtA* can be complemented by providing a full-length copy of *hrtA* in trans (Fig. 1B). HrtA is an ATPase which provides the energy required for hemin-detoxification by its cognate permease HrtB [13],[14]. Based on the phenotype of the *ΔhrtA* mutant, we reasoned that strains producing a catalytically inactive HrtA would elaborate a similar secreted protein profile upon hemin exposure. To test this hypothesis, we transformed *ΔhrtA* with plasmids encoding HrtA proteins that are mutated in key residues in the conserved nucleotide-binding and hydrolysis motifs (K45A, G145A, G145T, E167Q) or partially catalytically active (R76A) [14]. Surprisingly, expression of catalytically inactive versions of HrtA complemented the secreted protein profile of hemin-exposed *ΔhrtA* (Fig. 1B). These strains expressed comparable levels of HrtA, eliminating expression level as a possible factor in this analysis (Fig. 1C). These data indicate that it is not the catalytic activity of the HrtAB system that is required to prevent up-regulation of the immunomodulatory proteins upon hemin exposure, but instead it is the physical presence of HrtA that is required to prevent this phenotype.

### The *hrtB* and the *hrtA* genes are transcriptionally linked

**Figure 2:**
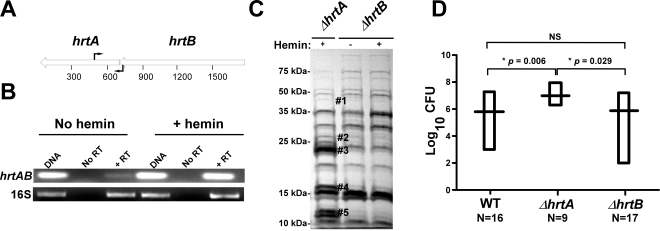
Loss of HrtB alone does not affect protein secretion or virulence in mice. (A) Diagrammatic representation of the *hrtAB* locus; the relative positions of the primers used for RT-PCR are indicated by black arrows. (B) Agarose gels showing the results of an RT-PCR experiment involving the *hrtAB* locus and the ribosomal 16S RNA as the loading control. Bacteria were grown ±1 µM hemin and RT-PCRs were done using DNA as the template (DNA), RNA with no reverse transcriptase (No RT) and RNA with reverse transcriptase (+RT). (C) Exoprotein profile of *ΔhrtA* + 1 µM hemin and *ΔhrtB* ±1 µM hemin. The # indicates the positions of proteins up-regulated under the indicated condition and the predicted identity of these proteins is as described in Figure 1. Positions of protein molecular mass markers in kilodaltons (kDa) are shown on the left side of the gel. (D) Bacterial multiplication in infected BALB/c mouse livers as measured by tissue homogenization, dilution, and colony formation on agar media 96 hours post infection. The horizontal bars represent the mean of log_10_CFU, and the boxes cover the range of log_10_CFU obtained in each group. * indicates statistically significant differences from *ΔhrtA* as determined by Student's *t* test with the indicated *p* values. NS indicates a non-statistically significant difference. N indicates the number of mice included in each group.

Based on the genomic organization of the *hrtAB* locus, it is expected that hemin exposure should increase HrtB expression in strains lacking *hrtA*. One potential explanation for the phenomenon described above is that HrtB acts as an unregulated pore in the absence of HrtA, sending a stress signal to the cell that leads to the up-regulation of immunomodulatory proteins. This model assumes that *hrtB* and *hrtA* are co-transcribed and over-expressed when *S. aureus* is exposed to hemin. To address this, RT-PCR experiments were performed using two primers that bind to the 3′-end of the *hrtB* gene and the 5′-end of the *hrtA* gene, respectively (Fig. 2A). The results of this experiment showed that *hrtA* and *hrtB* are indeed co-transcribed and transcription of the *hrt* operon increases upon hemin exposure (Fig. 2B). These results demonstrate that *hrtA* and *hrtB* comprise a heme-regulated bicistronic operon.

### Loss of HrtB alone does not affect protein secretion or virulence in mice

To further confirm that it is not inactivation of the HrtAB system that is responsible for the up-regulation of the immunomodulatory proteins, we constructed a strain containing a clean knock-out of *hrtB* (*ΔhrtB*). Upon examining the secreted protein profile of *ΔhrtB*, no notable changes were observed upon hemin exposure (Fig. 2C). We next sought to determine if the secreted protein profiles of *ΔhrtA* and *ΔhrtB* correlate with virulence levels in a murine model of systemic staphylococcal infection. Consistent with what has been reported previously, inactivation of *hrtA* leads to a significant increase in liver-specific virulence in this model [13]. However, when mice were infected with *ΔhrtB,* no significant difference was observed in liver colonization as compared to wildtype (Fig. 2D). These data link the secreted protein profile of hemin-exposed *ΔhrtA* to increased virulence and suggest that inactivation of HrtAB activity is not responsible for this hypervirulence.

### Hemin-exposed *ΔhrtA* has decreased membrane integrity

**Figure 3:**
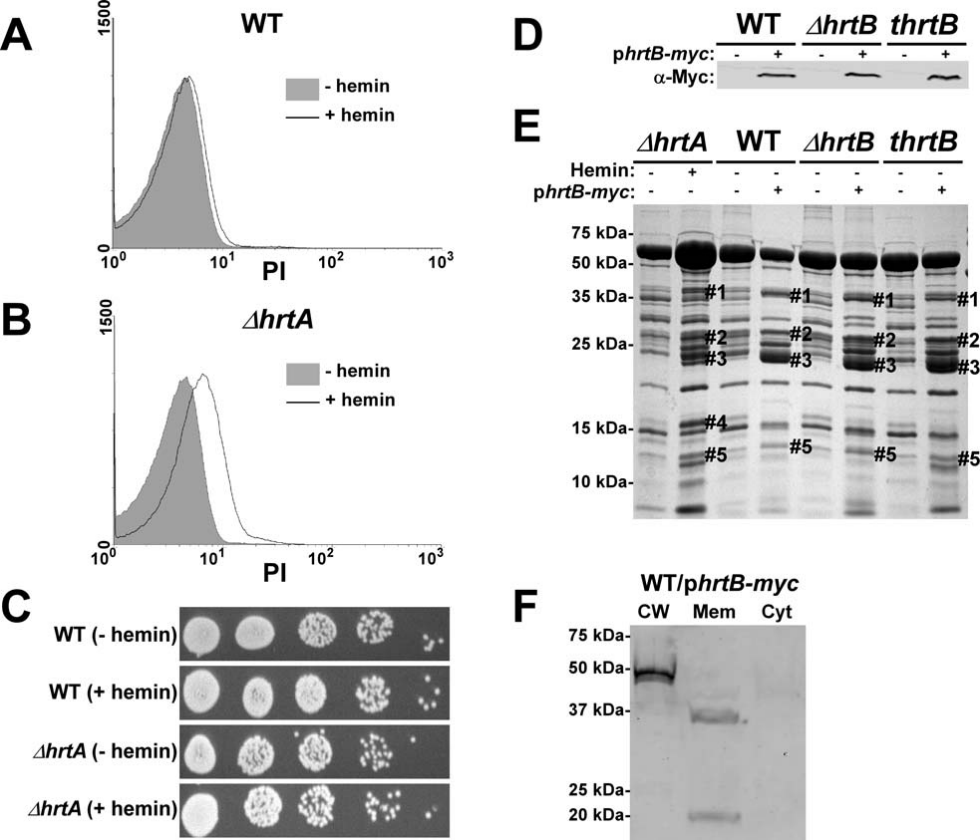
Over-expression of HrtB without hemin exposure mimics the effect of exposing *ΔhrtA* to hemin. (A and B) FACS analysis of wildtype (A) and *ΔhrtA* (B) cells incubated ±1 µM hemin and stained with propidium iodide (PI). The experiment was performed at least three times and a representative result is presented. (C) Viable counts of serial dilutions of cell suspensions analyzed in (A and B). (D) Immunoblot analysis of protoplast lysates of strains transformed with either control plasmid pOS1-plgt (−) or plasmid encoding constitutively expressed Myc-tagged HrtB p*hrtB-myc* (+). Immunoblots were analyzed as described in Figure 1C. (E) Exoprotein profile of the indicated strains were prepared and analyzed as in Figure 1. The # indicates the positions of proteins up-regulated under the indicated condition and the predicted identity of these proteins is as described in Figure 1. (F) Immunoblot analysis of cell fractions of WT/p*hrtB-myc*. Equivalent amounts of proteins were loaded from each fraction and the immunoblot was analyzed as described in Fig. 1C The reactive band in the cell wall fraction is the *S. aureus* protein A (Spa), which reacts non-specifically with anti-c-Myc antibody. Positions of protein molecular mass markers in kilodaltons (kDa) are indicated on the left side of panels D and E.

A potential explanation for the results obtained above is that hemin-exposed *ΔhrtA* over-expresses HrtB which localizes to the membrane as a dysregulated permease and causes membrane damage. In turn, this membrane damage is sensed by *S. aureus* leading to changes in the secreted protein profile. As an initial test of this hypothesis, both wildtype and *ΔhrtA* were grown in ±1 µM hemin and membrane integrity was determined by propidium iodide (PI) staining. PI is a membrane impermeant cationic stain that produces strong red fluorescence when bound to nucleic acids, and hence can be detected using FACS [22]. When wildtype *S. aureus* was exposed to hemin there was a small shift in PI staining indicating minor changes in membrane integrity (Fig. 3A). In contrast, *ΔhrtA* exposed to hemin exhibited a pronounced shift in PI staining indicative of substantial changes in membrane integrity (Fig. 3B). Cell death was excluded as a possible cause of the increased permeability of hemin exposed *ΔhrtA*, as hemin exposure at this concentration did not affect viability of the tested strains (Fig. 3C). These results indicate that exposure of *ΔhrtA* to 1 µM hemin compromises cell membrane integrity without affecting cellular viability.

### Hemin-independent over-expression of HrtB elicits a secreted protein profile that mimics that of hemin-exposed *ΔhrtA*

The data presented above are consistent with a model whereby HrtB expression in the absence of its cognate ATPase is responsible for increased secretion of immunomodulatory proteins. This phenotype can be complemented by catalytically inactive versions of HrtA suggesting that it is not HrtA ATPase activity but the physical presence of HrtA that prevents alterations in protein secretion. In keeping with this, it is predicted that discordant regulation of HrtB and HrtA would lead to a phenotype that mimics *ΔhrtA* exposed to hemin. To test this hypothesis, myc-tagged HrtB was constitutively expressed in wildtype, *ΔhrtB* and *thrtB* backgrounds. The three strains expressed comparable levels of HrtB as assessed by immunoblots using anti-c-Myc monoclonal antibody (Fig. 3D). Upon comparing the secreted protein profiles of these three strains in the absence of hemin exposure to matched strains harboring empty vector, we observed alterations in protein abundance that closely resemble those produced by hemin-exposed *ΔhrtA* (Fig. 3E). This result indicates that expressing disproportional ratios of HrtB:HrtA triggers a response similar to that obtained when HrtB is expressed in the absence of HrtA. To confirm that over-expressed HrtB localizes to the cell membrane, cell fractionation was performed on wildtype cells harboring the p*hrtB-myc* plasmid grown in the absence of hemin. When equivalent protein amounts of cellular fractions were immuno-blotted using monoclonal antibody against c-Myc, a reactive band with the predicted molecular mass (∼37 kDa) was detected exclusively in the membrane fraction (Fig. 3F). A second band migrating at approximately 20 kDa was detected in the membrane fraction, however the identity of this band in unknown at this point. These observations support a model whereby HrtB expression in the absence of HrtA acts as an unregulated pore resulting in the production of membrane stress and the up-regulation of immunomodulatory proteins.

### Membrane pore formation rather than generalized membrane damage in wildtype *S. aureus* mimics the effect of hemin on *ΔhrtA*

**Figure 4:**
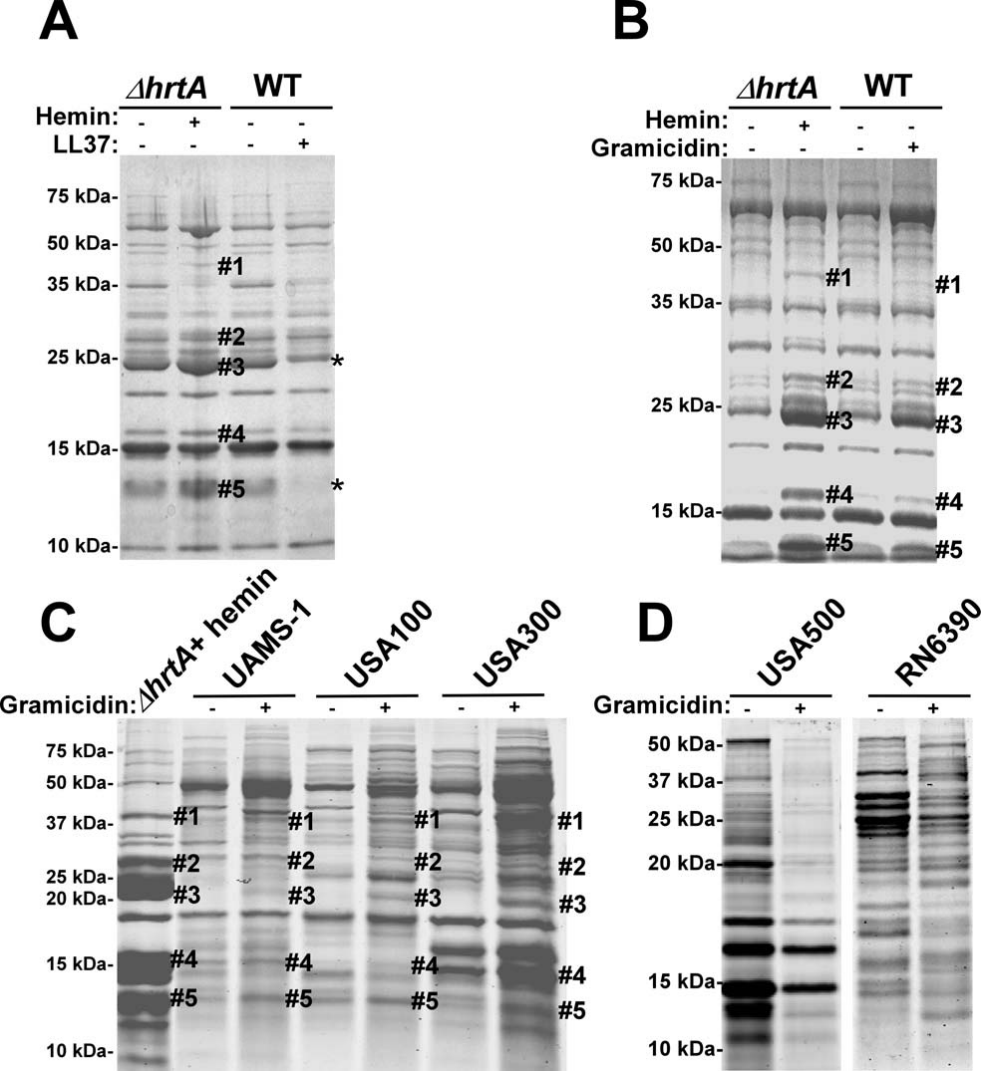
Membrane pore formation but not generalized membrane damage mimics the effect of hemin on *ΔhrtA*. (A) Exoprotein profile of *ΔhrtA* treated ±1 µM hemin and WT ±40 µg/ml of the antimicrobial peptide LL37. (B) Exoprotein profile of *ΔhrtA* treated ±1 µM hemin and WT ±16 µg/ml of the antimicrobial peptide gramicidin. (C) Exoprotein profile of *ΔhrtA* treated with 1 µM hemin and different staphylococcal strains ±32 µg/ml gramicidin. (D) Exoprotein profile of wildtype strains USA500 and RN6390 ±32 µg/ml gramicidin. Samples were prepared and analyzed as in Figure 1. The # indicates the positions of proteins up-regulated under the indicated condition and the predicted identity of these proteins is as described in Figure 1. The * indicates the positions of proteins down-regulated under the indicated condition. Positions of protein molecular mass markers in kilodaltons (kDa) are indicated on the left side of each panel.

To test if the secreted protein profile of hemin-exposed *ΔhrtA* is due to non-specific membrane damage, *S. aureus* was treated with sub-inhibitory concentrations of the anti-microbial peptide (AMP) LL37. LL37 elicits antimicrobial activity through membrane disruption caused by widespread random intercalation into the bacterial membrane [23]. We chose to use 40 µg/ml LL37 for these experiments as this concentration slightly inhibited *S. aureus* growth but permitted growth to cell densities that were similar to untreated cultures (data not shown). When the secreted protein profiles of *S. aureus* grown in the absence and presence of LL37 were compared to those of *ΔhrtA* grown in the absence and presence of 1 µM hemin, similar effects regarding the secreted proteins were not observed (Fig. 4A). None of the five bands that were visibly up-regulated in hemin-exposed *ΔhrtA* were up-regulated in wildtype exposed to LL37. In fact, some of the bands that were up-regulated in *ΔhrtA* exposed to hemin seemed to be down-regulated when wildtype was exposed to LL37 (Fig. 4A). This result indicates that membrane damage caused by LL37 treatment does not induce the same response within *S. aureus* as over-expression of the HrtB permease without its cognate ATPase. Therefore, non-specific membrane damage is not responsible for alterations in the secretome observed upon HrtA and HrtB dysregulation.

We next sought to test the impact of a pore-forming AMP on the staphylococcal secretome to more closely mimic the hypothesized membrane damage elicited upon HrtB over-expression. To this end, wildtype *S. aureus* was treated with sub-inhibitory concentrations of the pore-forming AMP gramicidin. The growth of *S. aureus* in the presence of 16 µg/ml gramicidin was slightly inhibited, but the treated cultures were able to reach similar optical densities to those of untreated cells (data not shown). Upon comparing the secreted protein profile of wildtype treated with gramicidin to that of *ΔhrtA* treated with hemin a remarkable conservation in the expression patterns was observed (Fig. 4B). Notably, all of the five bands that were up-regulated in hemin-exposed *ΔhrtA* were also up-regulated in gramicidin-treated wildtype, but to varying extents (Fig. 4B). This result supports the contention that dysregulated pore formation through the staphylococcal membrane leads to a specific alteration in protein secretion. Gramicidin exposure induces significant alterations in protein expression across a panel of *S. aureus* isolates (UAMS-1, USA100, USA300, USA500, and RN6390) (Fig. 4C & D). Notably, a subset of these strains (UAMS-1, USA100, and USA300) exhibit altered expression of proteins migrating to positions similar to those of the immunomodulatory proteins affected in the Newman strain (Fig. 4C). It is interesting to point out that not all strains analyzed were affected by gramicidin treatment equally. More specifically, USA500 and RN6390 do not seem to increase expression of the immunomodulatory proteins (based on migration pattern) upon gramicidin exposure despite exhibiting significant changes in protein expression (Fig. 4D). Taken together, these experiments suggest that treatment of distinct *S. aureus* strains with pore forming toxins produces changes in protein expression.

The *S. aureus* Aps/Gra system is a two-component system responsible for resistance to antimicrobial peptides suggesting a potential involvement in the response to gramicidin reported here [24],[25]. To evaluate the involvement of Aps/Gra in this response to gramicidin, we created a *S. aureus* strain inactivated for Aps/Gra (*ΔapsR*) and measured the impact of HrtB over-expression and gramicidin exposure on this strain. The secreted protein profile of *ΔapsR* did not differ noticeably from that of wildtype (Fig. S1A & B). These experiments revealed that the Aps/Gra system is not involved in the response to gramicidin or HrtB overexpression.

### The secretome of wildtype *S. aureus* treated with gramicidin resembles that of *ΔhrtA* exposed to hemin

**Figure 5:**
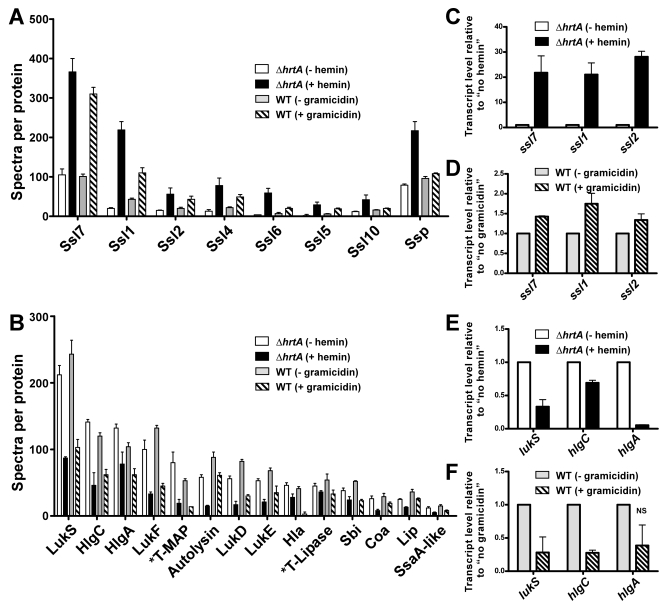
Treatment of *S. aureus* wildtype with gramicidin and *ΔhrtA* with hemin elicit similar secretomes. (A) Average number of spectra per protein of secreted proteins that were significantly up-regulated in *ΔhrtA* + 1 µM hemin and WT + 32 µg/ml gramicidin. (B) Average number of spectra per protein of secreted proteins that were significantly down-regulated under both conditions. The data presented are means of three independent samples and the error bars represent the standard deviation. The differences between *ΔhrtA* + hemin (black bars) and *ΔhrtA* - hemin (white bars) and WT + gramicidin (diagonally streaked bars) and WT - gramicidin (grey bars) are statistically significant in all the presented proteins with *p* values less than 0.05 as indicated by Student's *t* test. * T stands for truncated. (C-F) Results of quantitative real-time RT-PCR using RNA samples isolated from samples of *ΔhrtA* ± hemin and WT ± gramicidin. The levels of the transcripts were normalized using the levels of the ribosomal RNA 16S and the levels of the normalized transcripts from samples without treatment (either hemin or gramicidin) were used as calibrators. The data presented are the means of two independent experiments with each done in triplicate and the error bars represent the standard deviation. The differences between *ΔhrtA* + hemin (black bars) and *ΔhrtA* - hemin (white bars) and WT + gramicidin (diagonally streaked bars) and WT - gramicidin (grey bars) are statistically significant in all the presented transcripts with *p* values less than 0.05 as indicated by Student's *t* test except the one data point marked with NS.

Gel based comparisons of protein secretion between gramicidin-treated wildtype and hemin-exposed *ΔhrtA* suggest that these distinct but similar stressors lead to analogous alterations in the staphylococcal secretome (Figs. 3E and 4B). In an effort to increase the sensitivity and resolution of this comparison beyond Coomassie blue-stained SDS-PAGE analysis, a mass spectrometry-based approach known as shotgun proteomics (see Materials and Methods) was employed. Using shotgun proteomics we determined the proteomes of the culture supernatants of *ΔhrtA* grown in the presence and absence of hemin and wildtype *S. aureus* grown in the presence and absence of gramicidin. The analysis was performed on three independent samples from each condition. Quantitation of the proteins in each sample was performed using spectral counting of tandem spectra acquired for each protein normalized to the total number of spectra detected in the same sample. Analysis of the culture supernatants of *ΔhrtA* with and without hemin revealed the presence of at least 137 proteins exhibiting an average of at least 2 spectra in the three samples analyzed. Among these, 21 proteins were significantly up-regulated and 50 proteins were down-regulated in the presence of hemin (Table S2). Analysis of known secreted proteins demonstrated that there were 12 proteins significantly up-regulated and 18 proteins significantly down-regulated between these conditions (Tables 1 & 2, Figs. 5A & B). In the case of wildtype grown in the presence and absence of gramicidin, 101 proteins were detected among which 32 proteins were significantly up-regulated and 29 were down-regulated (Table S3). Among the 12 secreted proteins that were up-regulated in *ΔhrtA* exposed to hemin, 8 proteins (i.e. 75%) were also up-regulated in WT exposed to gramicidin (Table 1 and Fig. 5A). All 8 of these proteins have potential immunomodulatory functions including seven Staphylococcal Superantigen-Like proteins (Ssl1, Ssl2, Ssl4, Ssl5, Ssl6, Ssl7, and Ssl10) and the extracellular matrix and plasma binding protein (Ssp, also known as Emp). In addition three other Ssls (Ssl9, Ssl8 and Ssl3) were up-regulated under both conditions but their up-regulation did not reach the level of significance in wildtype treated with gramicidin (Table 1).

**Table 1:** Secreted proteins up-regulated in (*ΔhrtA* + hemin) and (WT + gramicidin).

Protein	*ΔhrtA* - hemin		*ΔhrtA* + hemin		*p*-value^3^	WT - gramicidin		WT + gramicidin		*p*-value^3^
	Mean^1^	SD^2^	Mean^1^	SD^2^		Mean^1^	SD^2^	Mean^1^	SD^2^	
**Ssl7**	105	15	366	34	0.0003	101	6	310	17	0.0000
**Ssl1**	20	2	219	21	0.0001	43	3	110	13	0.0009
**Ssl2**	15	1	56	16	0.0124	20	3	43	8	0.0088
**Ssl4**	13	4	78	19	0.0039	22	2	49	6	0.0022
**Ssl6**	4	0	59	12	0.0014	7	3	20	3	0.0066
**Ssl5**	2	3	29	7	0.0038	6	1	19	2	0.0011
**Ssl10**	12	1	42	12	0.0127	16	1	20	1	0.0146
**Ssp**	79	3	217	23	0.0005	96	5	108	2	0.0228
**Ssl8**	8	1	34	9	0.0090	10	1	20	7	0.0660
**Ssl3**	7	2	30	5	0.0026	12	1	14	2	0.2429*
**Ssl9**	6	1	53	15	0.0056	11	2	12	2	0.8676*
**FnbA**	2	1	7	1	0.0019	3	1	1	1	0.0477¥

1 Mean of the number of spectra detected in the three samples analyzed for this condition.

2 Standard deviation among the number of the spectra detected in the three samples analyzed for this condition.

3 *p*-value as obtained by applying Student's *t*-test.

*: The up-regulation of these proteins in the presence of gramicidin was not statistically significant.

**¥:** This protein was significantly down-regulated in the presence of gramicidin.

**Table 2:** Secreted proteins down-regulated in (*ΔhrtA* + hemin) and (WT + gramicidin).

Protein	*ΔhrtA* - hemin		*ΔhrtA* + hemin		*p*-value^3^	WT - gramicidin		WT + gramicidin		*p*-value^3^
	Mean^1^	SD^2^	Mean^1^	SD^2^		Mean^1^	SD^2^	Mean^1^	SD^2^	
**LukS**	212	14	87	2	0.0001	243	21	103	12	0.0005
**HlgC**	141	4	46	19	0.0011	120	5	62	8	0.0004
**HlgA**	132	6	78	18	0.0076	104	6	62	9	0.0028
**LukF**	100	14	33	3	0.0013	132	4	45	4	0.0000
**Truncated MAP**	80	16	19	6	0.0035	53	3	14	0	0.0000
**Autolysin**	58	4	15	1	0.0000	88	8	61	4	0.0057
**LukD**	56	4	17	5	0.0005	82	3	30	2	0.0000
**LukE**	53	3	21	4	0.0003	68	4	35	10	0.0060
**Hla**	46	4	28	5	0.0091	41	3	3	3	0.0001
**Truncated lipase**	45	4	36	2	0.0280	54	9	33	6	0.0265
**Sbi**	38	4	24	5	0.0237	52	1	23	2	0.0000
**Coa**	26	4	8	2	0.0028	29	5	19	2	0.0343
**Lip**	25	1	13	1	0.0004	36	4	26	1	0.0159
**SsaA-like**	12	2	5	1	0.0080	15	2	8	1	0.0151
**NAMLA amidase^ψ¥^**	18	5	4	1	0.0126	8	2	1	1	0.0105
**5′-nucleotidase** ¥	150	14	93	2	0.0024	148	13	119	11	0.0395
**SspB**	8	1	1	1	0.0009	6	1	3	2	0.0764*
**CHIPS**	169	20	108	9	0.0088	164	11	151	33	0.5285*

1 Mean of the number of spectra detected in the three samples analyzed for this condition.

2 Standard deviation among the number of the spectra detected in the three samples analyzed for this condition.

3 *p*-value as obtained by applying Student's *t*-test.

*: The down-regulation of these proteins in the presence of gramicidin was not statistically significant.

**ψ:** NAMLA stands for N-acetylmuramoyl-L-alanine amidase domain protein.

**¥:** This protein contains a probable N-terminal signal peptide sequence.

Among the 18 secreted proteins that were down-regulated in *ΔhrtA* exposed to hemin, 16 proteins (i.e. 89%) were also significantly down-regulated in wildtype treated with gramicidin (Table 2 and Fig. 5B). This group of proteins included several hemolysins, leukotoxins, and other known virulence factors. Two additional proteins (SspB and CHIPS) that were significantly down-regulated in *ΔhrtA* exposed to hemin were also down-regulated in wildtype exposed to gramicidin but their down-regulation did not reach the level of significance (Table 2). Taken together, these results demonstrate that pore formation through gramicidin treatment or permease dysregulation results in alterations in the staphylococcal exoprotein profile highlighted by an increased abundance of immunomodulatory proteins with known anti-neutrophil functions [19],[26],[27].

### Alterations in protein secretion upon pore formation occur at the transcriptional level

In order to determine whether the changes observed in the secretomes of *ΔhrtA* treated with hemin and wildtype treated with gramicidin are occurring at the transcriptional level, quantitative real-time RT-PCR was performed on a representative sample of genes. Upon testing the transcript levels of *ssl7*, *ssl1*, and *ssl2* as examples of genes encoding proteins that increase abundance upon pore formation, we noted a significant up-regulation in the level of all three transcripts in both *ΔhrtA* treated with hemin and wildtype treated with gramicidin (Figs. 5C & D). The fold up-regulation in *ΔhrtA* treated with hemin was higher than that observed in wildtype exposed to gramicidin. Further, upon testing the transcript levels of *lukS*, *hlgC*, and *hlgA* as examples of genes encoding proteins that decrease abundance upon pore formation, there was a significant down-regulation in the level of all three transcripts in *ΔhrtA* treated with hemin. This down-regulation was only significant in *lukS* and *hlgC* in wildtype treated with gramicidin (Figs. 5E & F). These findings demonstrate that the alterations in protein abundance that occur upon HrtB dysregulation or gramicidin treatment are occurring transcriptionally, consistent with the notion that *S. aureus* regulates an anti-neutrophil response upon sensing membrane damage elicited by pore forming toxins.

### The hypervirulence of strains lacking *hrtA* is due to the increased expression and secretion of Ssl1-11 and Ssp

**Figure 6:**
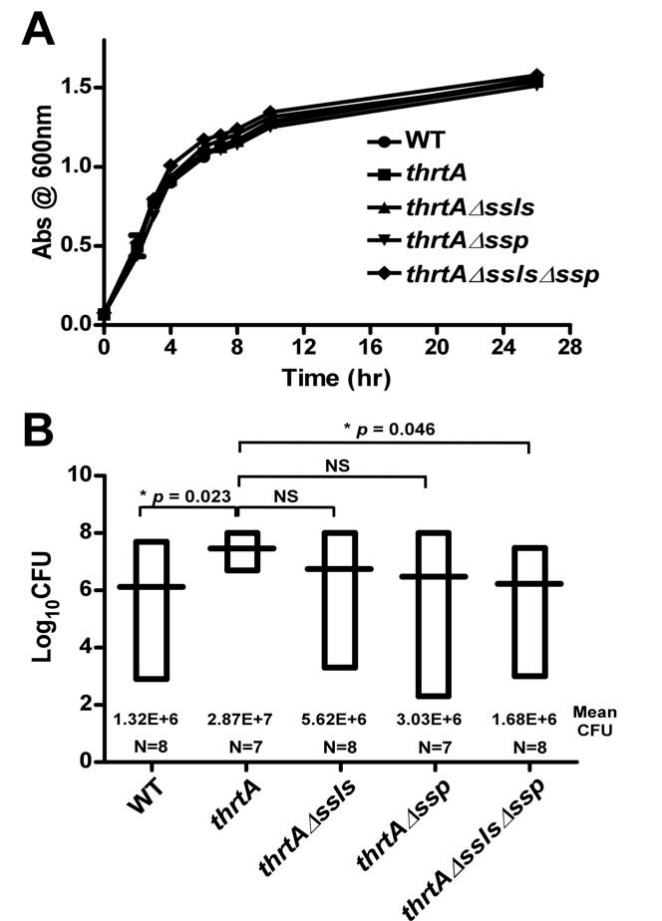
Ssls1-11 and Ssp are responsible for the liver-specific hyper-virulence of *hrtA* mutants. (A) Growth curve analysis of wildtype, *thrtA*, *thrtAΔssls*, *thrtAΔssp*, and *thrtAΔsslsΔssp*. Overnight cultures were diluted 1:20 in TSB and the absorbance at 600 nm was recorded at the indicated time points. A representative growth curve is presented. (B) Bacterial multiplication in infected BALB/c mice livers as measured by tissue homogenization, dilution, and colony formation on agar media 96 hours post infection. The horizontal bars represent the mean of log_10_CFU, and the boxes cover the range of log_10_CFU obtained in each group. * indicates statistically significant differences from *thrtA* as determined by Student's *t* test with the indicated *p* values. NS indicates a non-statistically significant difference. N indicates the number of mice included in each group.

Strains lacking *hrtA* exhibit liver-specific hypervirulence and increased secretion of immunomodulatory proteins such as Ssls and Ssp. To determine if the increased secretion of these immunomodulatory proteins is responsible for the hypervirulence of *hrtA* mutants, we created strains lacking *ssl1-11* or *ssp* in a *thrtA* background (*thrtAΔssls, thrtAΔssp*, *thrtAΔsslsΔssp).* Next we assessed the liver-specific virulence of *S. aureus* wildtype, *thrtA*, *thrtAΔssls, thrtAΔssp*, and *thrtAΔsslsΔssp* in the systemic model of staphylococcal infection described above. As expected, *thrtA* exhibited increased virulence in these studies as compared to wildtype. Inactivation of either *ssl1-11* or *ssp* reduced the virulence of *thrtA* to levels approximately equivalent to wildtype despite the fact that these mutations had no adverse effect on growth *in vitro* (Fig. 6). Inactivation of *ssp* had a more pronounced effect on the hypervirulence of *thrtA* as compared to *ssl1-11* underscoring the significant contribution of Ssp to the hypervirulence of *thrtA*. When the mutations of the *ssls* were combined with that of the *ssp* in the *hrtA* mutant background, the hypervirulence of the *hrtA* mutant was significantly reduced almost to the level of wildtype (Fig. 6). Taken together, these data demonstrate that the hypervirulence of *hrtA* mutants is due to the increased expression of Ssl1-11 and Ssp in response to dysregulated pore formation.

## Discussion

**Figure 7:**
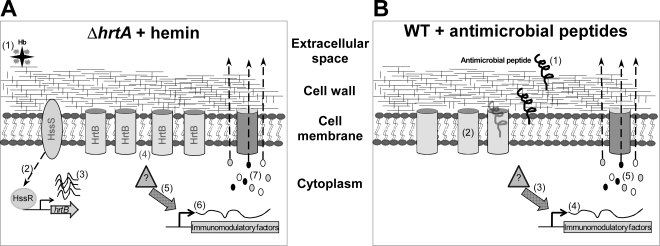
Membrane damage triggers immunomodulatory proteins secretion. (A) When *ΔhrtA* is exposed to hemin ^(1)^ the HssS/R system is activated ^(2)^ leading to over-expression of the *hrtB* gene ^(3)^ causing membrane damage by the over-expressed HrtB proteins acting as unregulated pores ^(4)^. This in turn activates an internal signal ^(5)^ that turns on the expression ^(6)^ and secretion ^(7)^ of the immunomodulatory proteins. (B) When WT *S. aureus* is attacked by antimicrobial peptides ^(1)^ that form pores in the membrane ^(2)^, this in turn activates an internal signal ^(3)^ that turns on the expression ^(4)^ and secretion ^(5)^ of immunomodulatory proteins.

*S. aureus* HrtAB is an ABC-type transport system that is essential for alleviating hemin toxicity [13],[14]. Upon hemin exposure HrtAB is up-regulated approximately 45-fold [28]. Inactivation of *hrtA* results in liver specific hyper-virulence that is associated with a hemin-induced over expression of immunomodulatory proteins [13]. Here we propose a revised model to explain the hyper-virulence of *S. aureus ΔhrtA* as summarized in Fig. 7A; when *ΔhrtA* is exposed to hemin, the HssRS system is activated leading to the over-expression of HrtB in the cell membrane without its cognate ATPase. In addition to an inability to relieve heme-toxicity [13], this mutant experiences membrane stress caused by the over-expressed HrtB permease acting as an unregulated pore. This membrane stress is sensed by *S. aureus* through an as-yet-unidentified mechanism, leading to over-expression and secretion of several immunomodulatory molecules. The combined action of these immunomodulatory proteins inhibits neutrophil migration to the site of infection enabling *ΔhrtA* to exhibit hypervirulence during liver colonization. This model is supported by the observation that *S. aureus* exposed to a pore forming AMP exhibits a similar secreted protein profile to hemin-exposed *ΔhrtA*, potentially providing a physiologically relevant explanation for the existence of this response (Fig. 7B). When *S. aureus* senses membrane damage in the form of pore formation, the organism appears to over-express and secrete immunomodulatory molecules that interfere with the recruitment of phagocytic cells to the site of infection. The observation that strains lacking *hssR* also exhibit hypervirulence suggest that disruption of HssRS signaling impacts HrtAB expression in a way that leads to a similar form of membrane damage [13].

The precise mechanism by which HrtB over-expression leads to membrane stress is not completely understood, but lack of expression of the HrtA protein may lead to misfolding of its cognate permease (HrtB), or lock the HrtB pore in a conformation that no longer allows the passage of its substrate, and/or allows the passage of ions involved in maintaining membrane potential. These possibilities are in accordance with the observation that over-expression of membrane proteins usually leads to reduced growth rates that are generally assumed to be due to negative effects on membrane integrity [29]. An indication that *ΔhrtA* exposed to hemin experiences membrane damage is provided by the observation that this strain showed increased permeability to propidium iodide (Fig. 3B) [22].

During infection, *S. aureus* faces an array of membrane damaging peptides and small proteins that perturb the cell membrane leading to cell leakage and eventually death [30],[31]. In fact, mammalian cells increase the production of several AMPs upon sensing *S. aureus* and its components [31],[32],[33]. In general, AMPs exert their antimicrobial activity through pore formation or membrane barrier disruption, however some AMPs mediate their antibacterial activity through altering septum formation or inhibiting cell-wall, nucleic-acid, or protein synthesis [34],[35],[36]. Few AMPs have been described that are considered channel-forming peptides that act through what is known as the “barrel-stave” mechanism [35],[37]. On the other hand, several AMPs, including the widely expressed human LL37, have been described to perturb membranes through what is known as the “carpet” or “detergent like” mechanism [23],[37].

To counter the bactericidal effects of AMPs, bacteria express regulatory systems designed to sense these innate immune threats to generate an anti-AMP response. Among these systems, the Gram negative PhoPQ system is the best characterized [38]. Recently, the staphylococcal counterpart of the PhoPQ system was identified [25],[39],[40]. The Aps/Gra system is a three component bacterial sensing system that senses AMPs to induce a resistance response that includes modification of cell surface components and increased expression of putative AMP transporters [25],[39],[40]. The Aps/Gra system is not involved in the phenotypes reported here, implying the existence of an additional system responsible for sensing membrane damage in *S. aureus* [40],[41],[42]. The *S. aureus* immunomodulatory factors described here were found to be up-regulated upon exposure to the pore-forming peptide gramicidin which is produced by *Bacillus brevis*. In contrast, this response is not elicited upon exposure to the membrane-disintegrating mammalian peptide LL37. Considering the apparent specificity of this secreted protein response to inhibit neutrophil migration, it is likely that as-yet-unidentified channel-forming AMPs elicit this response during mammalian infection.

*S. aureus* regulation of virulence factors in response to phagocytes and phagocytosis-related signals have been previously reported and the response varies according to the setting of the experiment and the strain tested. For instance, the *S. aureus* virulence regulator Sae is highly activated upon exposure to α-defensins and H_2_O_2_ but not LL37 [43]. When *S. aureus* strain SG511 was exposed to human *β*-defensin 3, the only pathogenic factors that were significantly up-regulated were *fmtA* and *sdrE* [44]. In a more comprehensive study [45], different *S. aureus* strains were phagocytosed by human PMNs and transcriptome analyses of these bacteria showed up-regulation of genes encoding multiple virulence factors such as *hlgA*, *hlgB*, *hlgC,* extracellular matrix and plasma binding protein (*ssp*), staphylocoagulase, and clumping factor. Interestingly, genes encoding several toxins, such as *ssl7*, *ssl11*, *ssl10* (*set14*), *lukD*, and *lukE* were up-regulated only in strains causing community acquired (CA) infections. When the CA-MRSA strain MW2 was exposed to neutrophil azurophilic granule proteins [46], there was an up-regulation of genes encoding numerous virulence factors including hemolysins (*hla*, *hld*, *hlgA*, *hlgB*, and *hlgC*) and leukotoxins (*lukS* and *lukF*) in addition to several immunomodulatory toxins belonging to the Ssl group (*ssl7*, *ssl1* and *ssl10*). In this manuscript we report that the *S. aureus* strain Newman response to either over-expression of the HrtB permease or the pore-forming AMP gramicidin is different from previous reports. We have observed up-regulation of the immunomodulatory Ssl exoproteins (Ssl7, 1, 2, 4, 6, 5, and 10) and extracellular matrix and plasma binding protein (Ssp) while simultaneously observing a down-regulation of several hemolysins, cytotoxins and other secreted virulence proteins. As observed with *S. aureus* strain Newman, we found that UAMS-1, USA100, USA300, USA500 and RN6390 undergo profound changes in protein expression upon exposure to gramicidin (Fig. 4C and D). A subset of these strains (UAMS-1, USA100, and USA300) exhibit altered expression of proteins migrating at the predicted size for the immunomodulatory proteins affected in the Newman strain. It is interesting to point out that not all strains analyzed were affected by gramicidin treatment equally. More specifically, USA500 and RN6390 do not seem to increase expression of the immunomodulatory proteins upon gramicidin exposure despite exhibiting significant changes in protein expression (Fig. 4D). From these data, we conclude that *S. aureus* alters exoprotein expression in response to pore-forming toxins.

The Ssl proteins have sequence and structural homology with superantigens; however they lack the superantigenic activity [19]. Functions have yet to be ascribed to each of the Ssl proteins, but those with known functions have been shown to have an immunomodulatory effect. For instance Ssl7 binds IgA and complement C5, blocking IgA-FcR interactions and complement activation [26]. Both Ssl5 and Ssl11 inhibit neutrophil rolling while Ssl5 was recently shown to inhibit leukocyte activation by chemokines and anaphylatoxins [27],[47]. In addition, Ssl10 inhibits CXCL12-induced human tumor cell migration [48]. However, very little is known about the regulation of these exotoxins [19]. It was shown that *ssl4* (*set9*) is up-regulated in a SarA-dependent manner while *ssl3* (*set8*) is up-regulated in an *agr*-dependent manner [49]. Combining these observations with the fact that many of the virulence factors that are down-regulated in our experiments such as Hla, HlgABC, Lip, and Map are also regulated through these two major *S. aureus* virulence regulators (*agr* and SarA) [49], it is tempting to speculate that one or both of these regulators may play a role in orchestrating the immunomodulatory program elicited by membrane damage caused by pore formation.

In conclusion, the data presented here shed light on a potentially new virulence regulatory circuit in *S. aureus* that can modulate the immune response in the host and the components of such a circuit represent potential therapeutic targets. The significance of this circuit stems from the fact that an inducer of this program, the antimicrobial agent gramicidin, is a component in preparations that have shown efficacy against *S. aureus* colonization and infections [50],[51]. The identification of the factors involved in transducing the signal from the membrane which activates the expression and secretion of these immunomodulatory factors is critical for the full understanding of this program and is the focus of future research.

## Materials and Methods

### Ethics statement

All procedures involving animals were approved by Vanderbilt University's Institutional Animal Care and Use Committee (IACUC). All animal experiments were performed in accordance to NIH guidelines, the Animal Welfare Act, and US federal law.

### Bacterial strains, plasmids and growth conditions

*Staphylococcus aureus* clinical isolate Newman [52] was used in all experiments as the wildtype strain (unless explicitly stated) and mutants were generated in its background. Isogenic mutants lacking the *hrtA* and *hssR* gene were previously described [13]. A strain containing a transposon insertion into *hrtB* (*thrtB*) was obtained from the Phoenix (N) library clone PhiNE 05560 (SAV2360) [53]. Plasmids expressing WT and mutated *hrtA* genes were described previously [14]. *S. aureus* were grown on tryptic soy broth (TSB) solidified with 1.5 % agar at 37°C or in TSB with shaking at 180 rpm, unless otherwise indicated. When appropriate TSB was supplemented with chloramphenicol at a final concentration of 10 µg/ml. *Escherichia coli* were grown in Luria broth (LB) and when needed, the media were supplemented with ampicillin at a final concentration of 100 µg/ml.

### Construction of mutants

#### Construction of the *hrtB* deletion mutant strain

The *ΔhrtB* mutant lacking the *hrtB* gene was constructed using established methods [54]. Briefly, a PCR amplicon containing the first 12 bp of the *hrtB* ORF together with approximately 600 bp upstream of the *hrtB* ORF was amplified using primers AA431 (5′-GGGGACCACTTTGTACAAGAAAGCTGGGTTAACGTCATCGTTTTA-3′) and AA407 (5′-GCACCTCCAATTGCTTCGATCGCTAATTTCATATCGATTCACTT-3′). Another amplicon containing the last 24 bp of the *hrtB* ORF together with approximately 750 bp downstream of the *hrtB* ORF was amplified using primers AA404 (5′-ATCGAAGCAATTGGAGGTG-3′) and AA428 (5′-GGGGACAAGTTTGTACAAAAAAGCAGGCTGCCTAAGAACTTAATG-3′). The first amplicon contained in its 3′-end 20 bp that are complementary to the first 20 bp in the 5′-end of the second amplicon. Both amplicons were used together as a template in a second cycle of PCR using primers AA431 and AA428. The resultant PCR product was recombined into pKOR1 [54] and used for allelic replacement into Newman as described [54].

#### Construction of the *ssl* deletion mutant strain

The *S. aureus* mutant strain lacking *ssls 1-11* (*Δssl1-11*) was constructed using the pKOR-1 plasmid as described by [54]. Briefly, sequences flanking the *ssl* locus were PCR amplified with primers VJT160 (5′-GGGGACAAGTTTGTACAAAAAAGCAGGCTAATAGTCCTCTTGCTCCTGC-3′) and VJT172 (5′-TCCCCCCGGGTTCAGACACAAAACAGACATC-3′) for the upstream fragment and primers VJT124 (5′-GGGGACCACTTTGTACAAGAAAGCTGGGTTAAAACCATACCTTCATCATCC- 3′) and VJT173 (5′-TCCCCCCGGGGAGTTAATCACTGACTTCTAC-3′) for a downstream fragment. The PCR amplicons were cloned into pCR2.1 (Invitrogen), and then digested with XmaI and assembled into pCR2.1. A PCR amplicon of the joined DNA fragment was recombined into pKOR1 resulting in the pKOR-1Δ*ssl1-11*plasmid. Deletion of the *ssl* locus in Newman was achieved by allelic replacement with pKOR-1Δ*ssl1-11*.

#### Construction of the *hrtA::bursa ssl* double mutant strain

An erythromycin cassette insertion mutant of *hrtA* was obtained from the Phoenix (N) library (*thrtA*) [13],[53]. The *hrtA::erm* mutation was transduced into Newman lacking *ssls* (*Δssl1-11*) with phage Φ85 [10].

#### Construction of the *ssp* deletion mutant strain

Primers AA547 (5′- GGGGACAAGTTTGTACAAAAAAGCAGGCTGAAAACACCTGTAGTGTCATTAGA-3′) and AA543 (5′- AACCCGGGGCTCATAGTTAAAACTAATAA-3′, XmaI site underlined) were used to amplify the upstream region of the *ssp* ORF (NWMN_0758) and primers AA545 (5′- CACCCGGGGTATAAAAATTGGCACTAAGT-3′, XmaI site underlined) and AA548 (5′- GGGGACCACTTTGTACAAGAAAGCTGGGTGGTTTGCTTAATGTGTTAACTTTT-3′) were used to amplify the region downstream of the *ssp* ORF. Both PCR amplicons were digested with XmaI then ligated, PCR-amplified and ligated into pCR2.1 vector. The resulting plasmid was digested with XmaI and ligated to the non-polar spectinomycin cassette from plasmid pSL60-1[55]. The PCR fragment containing the flanking regions and the spectinomycin resistance cassette was then amplified and recombined into pKOR1. The *Δssp::spc* mutant was constructed in strain Newman as described above then transduced into the *thrtA* mutant and *thrtA. Δssls* mutant background.

#### Construction of the *apsR* deletion mutant strain

The *ΔapsR* mutant lacking the *apsR* gene (NWMN_0628) encoding the response regulator of the Aps/Gra system was constructed using a similar approach to that described above for the construction of the *ΔhrtB* and *Δssl1-11* mutants.

### Construction of a plasmid over-expressing HrtB

For the construction of a plasmid constitutively expressing Myc-tagged HrtB, a PCR amplicon was made containing the *hrtB* ORF into which the sequence encoding a c-Myc-epitope (EQKLISEEDL) was inserted just prior to the stop codon using the primers DS218 (5′- GGGGCATATGAAATTAGCGATAAAAGAG-3′, NdeI site underlined) for the 5′-end and AA494 (5′- TAGATCTTCTTCAGATATCAGTTTCTGTTCGCCGCCTTCTGCACCTCCAATTGCTTCGATAGGATCCACTTT-3′) together with AA495 (5′-CCTCGAGTTATAGATCTTCTTCAGATATCAGTTT-3′, XhoI site underlined) for the 3′-end. The PCR product was then digested with both NdeI and XhoI and ligated into the *E. coli*/*S. aureus* shuttle vector pOS1-plgt [56] which had been digested with the same restriction enzymes. Ligation mixtures were then transformed into *E. coli* DH5α and the resultant plasmid was designated p*hrtB-myc*. After DNA sequence verification the plasmid was transformed into the restriction-deficient modification-positive *S. aureus* RN4220 [57] followed by transformation into the respective electrocompetent *S. aureus* strain using the protocol described [58].

### Cell fractionation

*S. aureus* cells were grown to mid-log phase and sedimented by centrifugation at 3,200×*g* for 10 min. The supernatants were removed and the pellet was suspended in 500 µl TSM (100 mM Tris-HCl (pH 7.0), 500 mM sucrose, 10 mM MgCl_2_) supplemented with 100 µg lysostaphin and incubated for 1 hr at 37°C. The resulting protoplasts were sedimented by centrifugation at 16,000×*g* for 15 min; the supernatant was collected [cell wall (CW) fraction], and the pellet was suspended in 500 µl membrane buffer (50 mM Tris-HCl (pH 7.0), 10 mM MgCl_2_, 60 mM KCl) and subjected to sonication. The membranes were sedimented by centrifugation at 100,000×*g* for 45 min. The supernatant was collected [cytoplasm (Cyt) fraction] and the pellet [membrane (Mem) fraction] was suspended in 200 µl membrane buffer. Equivalent amounts of protein from each fraction were separated by SDS-PAGE and analyzed by immunoblotting as described below.

### Measuring membrane integrity using propidium iodide staining

In order to monitor staphylococcal membrane integrity, bacterial cells were grown in Roswell Park Memorial Institute (RPMI) medium supplemented with 1% (wt/vol) Casamino Acids (CAS) ±1 µM hemin until the cultures reached mid-log phase (OD_600_ ∼0.4), washed and suspended in phosphate buffered saline (PBS) supplemented with 1% wt/vol bovine serum albumen (BSA). All samples were adjusted to the same OD_600_ and 50 µl aliquots corresponding to approximately 10^7^ cells were mixed with 2 µg of propidium iodide (PI) (Sigma) in 1 ml PBS/1%BSA and analyzed by fluorescence-activated cell sorting (FACS). Analysis was performed on a FACSCalibur system, (BD, Franklin Lakes, NJ) using Cell Quest Pro software (BD). Equal aliquots of each sample were serially diluted and plated on TSA for viability counting.

### Antimicrobial peptides and MIC determination

The AMP LL37 was obtained from Phoenix Pharmaceuticals, Inc (Burlingame, CA) and it was dissolved directly in RPMI/CAS. The AMP gramicidin was obtained from Sigma (St. Louis, MO) and dissolved in absolute ethanol to a final concentration of 10 mg/ml. MIC determination was done by incubating 10^5^ CFU/ml *S. aureus* with 2 fold serial dilutions of the tested peptide in RPMI/CAS at 37°C. The MIC was considered as the lowest concentration that prevented growth as indicated by lack of visible turbidity following incubation at 37°C for 24 hr.

### Analyses of secreted proteins

*S. aureus* cultures from a fresh streak on TSA plates were inoculated in 5 ml of RPMI/CAS overnight at 37°C. The overnight cultures were then used to inoculate a new 5 ml culture in a 15 ml concical tube to which hemin was added at final concentrations of 0.5, 1, or 2 µM. The AMP LL37 was added to a final concentration of 40 µg/ml and gramicidin was added to a final concentration of 16 or 32 µg/ml. As a negative control, cells were grown in RPMI/CAS with equivalent volume of ethanol (WT – gramicidin). All the cultures were allowed to grow overnight (∼18 hrs) then the samples were normalized to the same OD_600_. Bacterial cells were sedimented by centrifugation and proteins in the culture supernatants were precipitated using 10% (vol/vol) TCA at 4°C overnight (∼16 hrs). The precipitated proteins were sedimented by centrifugation, washed with absolute ethanol, dried, resuspended in 1X SDS-loading buffer, and boiled for 10 min. Proteins in the samples were resolved using 15% wt/vol SDS-PAGE, and stained with Coomassie blue. At times, we observed slight variations in protein expression from identical strains used in distinct experiments as shown in Figures 1, 2, 3, 4, and S1. This variation correlated with changes in the source of medium, suggesting that batch-to-batch variation in media preparations affects this phenotype. Despite these minor variations, similar patterns of protein expression were observed across all experiments.

### Shotgun proteomics analysis of *S. aureus* exoproteins

TCA precipitated proteins from filtered culture supernatants of *ΔhrtA* ±1 µM hemin and WT ±32 µg/ml gramicidin were prepared as described above. Higher concentration of gramicidin than what was used above was aimed to increase the sensitivity of the assay. Proteins were resuspended in 1X SDS-digestion buffer and loaded into 12% SDS-PAGE gel (without a stacking gel) and electrophoresed 2 cm into the gel. The gel was stained with Colloidal Blue (Invitrogen, Carlsbad, CA) then destained with distilled water over night. Proteins were then subjected to in-gel trypsin digestion and peptide extraction and the resulting peptides were then analyzed using a Thermo Finnigan LTQ ion trap instrument and separated as described [59]. Tandem spectra were acquired using a data dependent scanning mode with a one full MS scan (*m/z* 400–2000) followed by 9 MS-MS scans. The SEQUEST algorithm was then used to search the tandem spectra against the Newman strain of the *S. aureus* subset of the UniRef100 database. To determine false positive rates, the database was concatenated with the reverse sequences of all proteins in the database. The SEQUEST outputs were filtered through the ID Picker suite with a false positive ID threshold of 5% and proteins were required to be identified by 2 or more unique peptides. Protein reassembly from identified peptide sequences was done as previously described [60]. The number of spectra identified for each protein under a given condition were normalized to the number of total spectra detected in the same injection.

### RNA isolation and manipulation

*S. aureus* cultures where grown in RPMI/CAS with the appropriate additive to an OD_600_ ∼1.0. Cultures were then mixed with equal volume of 1∶1 ethanol: acetone mixture and frozen at -80°C. For RNA extraction, frozen samples were allowed to thaw on ice; cells were then sedimented and washed two times with TE buffer. Cells were broken mechanically using fastprep bead beater (MP Biomedicals, Solon, OH) then RNA was isolated using the RNeasy mini kit (Qiagen, Valencia, CA) according to the manufacturer's recommendation. On-column DNase digestion was performed and after RNA elution, the samples were cleaned from any residual DNA contamination using the MessageClean kit (GenHunter, Nashville, TN).

### RT-PCR and real time RT-PCR

RT-PCR was performed to determine if the *hrtB* and *hrtA* genes were transcriptionally linked. 2 µg of RNA isolated as described above was used as template for a reverse transcriptase reaction using random hexmer (Promega, Madison, WI) and the M-MLV reverse transcriptase (Promega). The synthesized cDNA was then used as a template for a PCR reaction using primers AA492 (5′-TCCTATCGAAGCAATTGGAGGTGC-3′) which binds near the 3′-end of the *hrtB* gene and AA493 (5′-TCCCAGAACCAGAGGCACCATTTA-3′) which binds near the 5′-end of the *hrtA* gene. Control reactions were carried out where DNA and RNA without reverse transcriptase treatment were used as templates. For real time RT-PCR, 50 ng, or 5 ng in case of 16S, of total RNA was reverse transcribed and PCR-amplified by the MultiScribe enzyme (Applied Biosytems, Foster City, CA) in the presence of sybr green PCR mix (Applied Biosytems,) using primers specific for each of the analyzed transcripts (Table S1). 16S RNA was utilized at 5 ng in order to prevent the reaction rapidly reaching saturation which would hinder normalization. The levels of all transcripts were normalized to the level of the ribosomal RNA 16S. The normalized transcript levels of the respective strain without treatment (either hemin or gramicidin) were used as calibrators. All samples were analyzed in triplicate and the data were then analyzed by the iQ5 standard edition software (Bio-rad, Hercules, CA) using the *^ΔΔ^*CT method.

### Immunoblots

*S. aureus* cultures were grown in RPMI/CAS overnight, cells were then treated with lysostaphin to digest the cell wall and the resulting protoplasts were pelleted and resuspended in BugBuster Protein Extraction Reagent (Novagen, Gibbstown, NJ) with proteinase inhibitor and sonicated for 10 s. Proteins in protoplasts were then resolved in 15% SDS-PAGE, transferred to nitrocellulose membranes, immunoblotted with 9E10 anti-C-Myc monoclonal antibody as a primary antibody and AlexaFluor-680-conjugated anti-mouse as secondary antibody. Membranes were then scanned using an Odyssey Infrared Imaging System (LI-Cor Biosciences, Lincoln, NE).

### Murine model of infection

Six- to eight-week-old BALB/c female mice (Jackson Laboratories, Bar Harbor, Maine) were infected retro-orbitally with approximately 1×10^7^ CFU of *S. aureus* strains. Ninety-six hours post-infection, mice were euthanized with CO_2_, livers were removed, homogenized in sterile PBS, serially diluted and plated on TSA for colony forming unit (CFU) counts. At least seven mice were infected with each strain and statistical analyses were performed using the Student's *t* test, where *p* values <0.05 were considered statistically significant.

## Supporting Information

Figure S1 The Aps/Gra system is not involved in the observed changes in the secreted proteins profile phenotype. (A) Exoprotein profile of wildtype strain and *ΔapsR* containing plasmids pOS1-plg and p*hrtB-myc*. (B) Exoprotein profile of wildtype strain and *ΔapsR* ±32 µg/ml gramicidin. The # indicates the positions of proteins up-regulated under the indicated condition and the predicted identity of these proteins is as described in Figure 1. Positions of protein molecular mass markers in kilodaltons (kDa) are indicated on the left side of each panel.
(0.66 MB TIF)

Table S1 Primers used for real time RT-PCR analysis.
(0.03 MB XLS)

Table S2 Proteins significantly changed in the culture supernatants of (*ΔhrtA* + hemin)compared to (*ΔhrtA*- hemin).
(0.05 MB XLS)

Table S3 Proteins significantly changed in the culture supernatants of (WT + gramicidin) compared to (WT - gramicidin).
(0.05 MB XLS)

## References

[1] Wertheim HF, Vos MC, Ott A, van Belkum A, Voss A (2004). Risk and outcome of nosocomial *Staphylococcus aureus* bacteraemia in nasal carriers versus non-carriers.. Lancet.

[2] Diekema DJ, Pfaller MA, Schmitz FJ, Smayevsky J, Bell J (2001). Survey of infections due to *Staphylococcus* species: frequency of occurrence and antimicrobial susceptibility of isolates collected in the United States, Canada, Latin America, Europe, and the Western Pacific region for the SENTRY Antimicrobial Surveillance Program, 1997-1999.. Clin Infect Dis.

[3] Klevens RM, Morrison MA, Nadle J, Petit S, Gershman K (2007). Invasive methicillin-resistant *Staphylococcus aureus* infections in the United States.. JAMA.

[4] Lowy FD (1998). *Staphylococcus aureus* infections.. N Engl J Med.

[5] DeLeo FR, Diep BA, Otto M (2009). Host defense and pathogenesis in *Staphylococcus aureus* infections.. Infect Dis Clin North Am.

[6] Bullen DJ, Griffiths E (1999). Iron and Infection: Molecular, Physiological and Clinical Aspects, 2nd Edition ed..

[7] Skaar EP, Gaspar AH, Schneewind O (2004). IsdG and IsdI, heme-degrading enzymes in the cytoplasm of *Staphylococcus aureus*.. J Biol Chem.

[8] Reniere ML, Torres VJ, Skaar EP (2007). Intracellular metalloporphyrin metabolism in *Staphylococcus aureus*.. Biometals.

[9] Pishchany G, Dickey SE, Skaar EP (2009). Subcellular localization of the *Staphylococcus aureus* heme-iron transport components IsdA and IsdB.. Infect Immun.

[10] Mazmanian SK, Skaar EP, Gaspar AH, Humayun M, Gornicki P (2003). Passage of heme-iron across the envelope of *Staphylococcus aureus*.. Science.

[11] Torres VJ, Pishchany G, Humayun M, Schneewind O, Skaar EP (2006). *Staphylococcus aureus* IsdB is a hemoglobin receptor required for heme iron utilization.. J Bacteriol.

[12] Stauff DL, Torres VJ, Skaar EP (2007). Signaling and DNA-binding activities of the *Staphylococcus aureus* HssR-HssS two-component system required for heme sensing.. J Biol Chem.

[13] Torres VJ, Stauff DL, Pishchany G, Bezbradica JS, Gordy LE (2007). A *Staphylococcus aureus* regulatory system that responds to host heme and modulates virulence.. Cell Host Microbe.

[14] Stauff DL, Bagaley D, Torres VJ, Joyce R, Anderson KL (2008). *Staphylococcus aureus* HrtA is an ATPase required for protection against heme toxicity and prevention of a transcriptional heme stress response.. J Bacteriol.

[15] Davidson AL, Chen J (2004). ATP-binding cassette transporters in bacteria.. Annu Rev Biochem.

[16] Higgins CF, Linton KJ (2004). The ATP switch model for ABC transporters.. Nat Struct Mol Biol.

[17] Holland IB, Blight MA (1999). ABC-ATPases, adaptable energy generators fuelling transmembrane movement of a variety of molecules in organisms from bacteria to humans.. J Mol Biol.

[18] Davidson AL, Dassa E, Orelle C, Chen J (2008). Structure, function, and evolution of bacterial ATP-binding cassette systems.. Microbiol Mol Biol Rev.

[19] Fraser JD, Proft T (2008). The bacterial superantigen and superantigen-like proteins.. Immunol Rev.

[20] Williams RJ, Ward JM, Henderson B, Poole S, O'Hara BP (2000). Identification of a novel gene cluster encoding staphylococcal exotoxin-like proteins: characterization of the prototypic gene and its protein product, SET1.. Infect Immun.

[21] Prat C, Bestebroer J, de Haas CJ, van Strijp JA, van Kessel KP (2006). A new staphylococcal anti-inflammatory protein that antagonizes the formyl peptide receptor-like 1.. J Immunol.

[22] Weller K, Lauber S, Lerch M, Renaud A, Merkle HP (2005). Biophysical and biological studies of end-group-modified derivatives of Pep-1.. Biochemistry.

[23] Porcelli F, Verardi R, Shi L, Henzler-Wildman KA, Ramamoorthy A (2008). NMR structure of the cathelicidin-derived human antimicrobial peptide LL-37 in dodecylphosphocholine micelles.. Biochemistry.

[24] Kraus D, Herbert S, Kristian SA, Khosravi A, Nizet V (2008). The GraRS regulatory system controls *Staphylococcus aureus* susceptibility to antimicrobial host defenses.. BMC Microbiol.

[25] Lai Y, Villaruz AE, Li M, Cha DJ, Sturdevant DE (2007). The human anionic antimicrobial peptide dermcidin induces proteolytic defence mechanisms in staphylococci.. Mol Microbiol.

[26] Langley R, Wines B, Willoughby N, Basu I, Proft T (2005). The staphylococcal superantigen-like protein 7 binds IgA and complement C5 and inhibits IgA-Fc alpha RI binding and serum killing of bacteria.. J Immunol.

[27] Bestebroer J, Poppelier MJ, Ulfman LH, Lenting PJ, Denis CV (2007). Staphylococcal superantigen-like 5 binds PSGL-1 and inhibits P-selectin-mediated neutrophil rolling.. Blood.

[28] Friedman DB, Stauff DL, Pishchany G, Whitwell CW, Torres VJ (2006). *Staphylococcus aureus* redirects central metabolism to increase iron availability.. PLoS Pathog.

[29] Wagner S, Bader ML, Drew D, de Gier JW (2006). Rationalizing membrane protein overexpression.. Trends Biotechnol.

[30] Gudmundsson GH, Agerberth B (1999). Neutrophil antibacterial peptides, multifunctional effector molecules in the mammalian immune system.. J Immunol Methods.

[31] Komatsuzawa H, Ouhara K, Yamada S, Fujiwara T, Sayama K (2006). Innate defences against methicillin-resistant *Staphylococcus aureus* (MRSA) infection.. J Pathol.

[32] Harder J, Bartels J, Christophers E, Schroder JM (2001). Isolation and characterization of human β-defensin-3, a novel human inducible peptide antibiotic.. J Biol Chem.

[33] Dinulos JG, Mentele L, Fredericks LP, Dale BA, Darmstadt GL (2003). Keratinocyte expression of human β−defensin 2 following bacterial infection: role in cutaneous host defense.. Clin Diagn Lab Immunol.

[34] Koo SP, Bayer AS, Yeaman MR (2001). Diversity in antistaphylococcal mechanisms among membrane-targeting antimicrobial peptides.. Infect Immun.

[35] Brogden KA (2005). Antimicrobial peptides: pore formers or metabolic inhibitors in bacteria?. Nat Rev Microbiol.

[36] Hale JD, Hancock RE (2007). Alternative mechanisms of action of cationic antimicrobial peptides on bacteria.. Expert Rev Anti Infect Ther.

[37] Shai Y (2002). Mode of action of membrane active antimicrobial peptides.. Biopolymers.

[38] Bader MW, Sanowar S, Daley ME, Schneider AR, Cho U (2005). Recognition of antimicrobial peptides by a bacterial sensor kinase.. Cell.

[39] Li M, Lai Y, Villaruz AE, Cha DJ, Sturdevant DE (2007). Gram-positive three-component antimicrobial peptide-sensing system.. Proc Natl Acad Sci U S A.

[40] Herbert S, Bera A, Nerz C, Kraus D, Peschel A (2007). Molecular basis of resistance to muramidase and cationic antimicrobial peptide activity of lysozyme in staphylococci.. PLoS Pathog.

[41] Novick RP (2003). Autoinduction and signal transduction in the regulation of staphylococcal virulence.. Mol Microbiol.

[42] Cheung AL, Bayer AS, Zhang G, Gresham H, Xiong YQ (2004). Regulation of virulence determinants in vitro and in vivo in *Staphylococcus aureus*.. FEMS Immunol Med Microbiol.

[43] Geiger T, Goerke C, Mainiero M, Kraus D, Wolz C (2008). The virulence regulator Sae of *Staphylococcus aureus*: promoter activities and response to phagocytosis-related signals.. J Bacteriol.

[44] Sass V, Pag U, Tossi A, Bierbaum G, Sahl HG (2008). Mode of action of human β−defensin 3 against *Staphylococcus aureus* and transcriptional analysis of responses to defensin challenge.. Int J Med Microbiol.

[45] Voyich JM, Braughton KR, Sturdevant DE, Whitney AR, Said-Salim B (2005). Insights into mechanisms used by *Staphylococcus aureus* to avoid destruction by human neutrophils.. J Immunol.

[46] Palazzolo-Ballance AM, Reniere ML, Braughton KR, Sturdevant DE, Otto M (2008). Neutrophil microbicides induce a pathogen survival response in community-associated methicillin-resistant *Staphylococcus aureus*.. J Immunol.

[47] Bestebroer J, van Kessel KP, Azouagh H, Walenkamp AM, Boer IG (2009). Staphylococcal SSL5 inhibits leukocyte activation by chemokines and anaphylatoxins.. Blood.

[48] Walenkamp AM, Boer IG, Bestebroer J, Rozeveld D, Timmer-Bosscha H (2009). Staphylococcal superantigen-like 10 inhibits CXCL12-induced human tumor cell migration.. Neoplasia.

[49] Dunman PM, Murphy E, Haney S, Palacios D, Tucker-Kellogg G (2001). Transcription profiling-based identification of *Staphylococcus aureus* genes regulated by the *agr* and/or *sarA* loci.. J Bacteriol.

[50] Fung S, O'Grady S, Kennedy C, Dedier H, Campbell I (2000). The utility of polysporin ointment in the eradication of methicillin-resistant *Staphylococcus aureus* colonization: a pilot study.. Infect Control Hosp Epidemiol.

[51] Schubert C, Moosa MR (2007). Infective endocarditis in a hemodialysis patient: a dreaded complication.. Hemodial Int.

[52] Duthie ES, Lorenz LL (1952). Staphylococcal coagulase; mode of action and antigenicity.. J Gen Microbiol.

[53] Bae T, Banger AK, Wallace A, Glass EM, Aslund F (2004). *Staphylococcus aureus* virulence genes identified by *bursa aurealis* mutagenesis and nematode killing.. Proc Natl Acad Sci U S A.

[54] Bae T, Schneewind O (2006). Allelic replacement in *Staphylococcus aureus* with inducible counter-selection.. Plasmid.

[55] Lukomski S, Hoe NP, Abdi I, Rurangirwa J, Kordari P (2000). Nonpolar inactivation of the hypervariable streptococcal inhibitor of complement gene (sic) in serotype M1 *Streptococcus pyogenes* significantly decreases mouse mucosal colonization.. Infect Immun.

[56] Bubeck Wardenburg J, Williams WA, Missiakas D (2006). Host defenses against *Staphylococcus aureus* infection require recognition of bacterial lipoproteins.. Proc Natl Acad Sci U S A.

[57] Novick RP (1991). Genetic systems in staphylococci.. Methods Enzymol.

[58] Schenk S, Laddaga RA (1992). Improved method for electroporation of *Staphylococcus aureus*.. FEMS Microbiol Lett.

[59] Tabb DL, Fernando CG, Chambers MC (2007). MyriMatch: highly accurate tandem mass spectral peptide identification by multivariate hypergeometric analysis.. J Proteome Res.

[60] Zhang B, Chambers MC, Tabb DL (2007). Proteomic parsimony through bipartite graph analysis improves accuracy and transparency.. J Proteome Res.

