# Supplementary figures and images for: Membrane Damage Elicits an Immunomodulatory Program in Staphylococcus aureus
Source: PLoS Pathog. 2010 Mar 12;6(3):e1000802. doi: 10.1371/journal.ppat.1000802 (PMC2837406; doi:10.1371/journal.ppat.1000802)

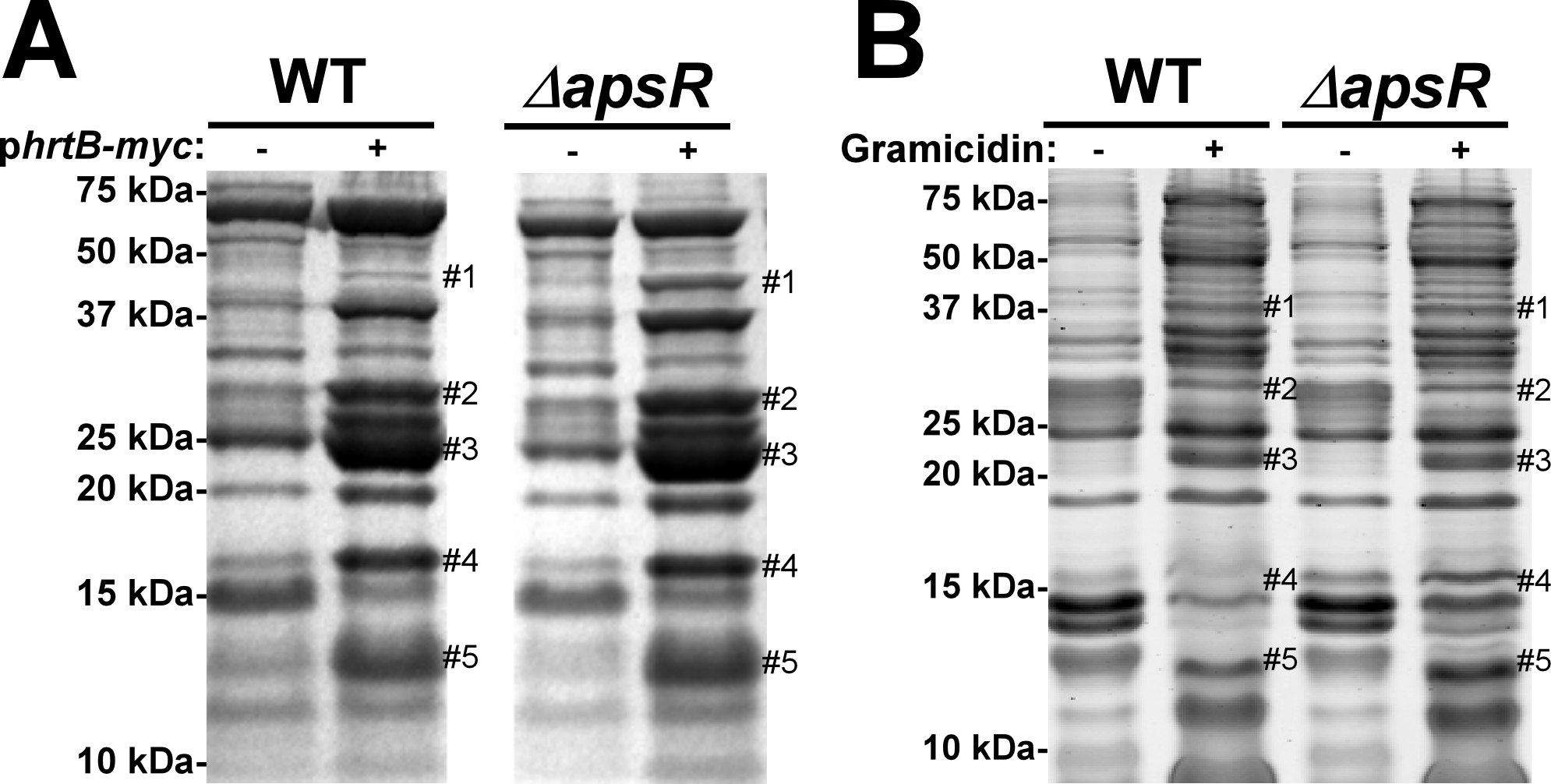

Supplement: Figure S1 — The Aps/Gra system is not involved in the observed changes in the secreted proteins profile phenotype. (A) Exoprotein profile of wildtype strain and ΔapsR containing plasmids pOS1-plg and phrtB-myc. (B) Exoprotein profile of wildtype strain and ΔapsR ±32 µg/ml gramicidin. The # indicates the positions of proteins up-regulated under the indicated condition and the predicted identity of these proteins is as described in Figure 1. Positions of protein molecular mass markers in kilodaltons (kDa) are indicated on the left side of each panel. (0.66 MB TIF) [file ppat.1000802.s001.tif]
